# Breed Distribution and Allele Frequencies of Base Coat Color, Dilution, and White Patterning Variants across 28 Horse Breeds

**DOI:** 10.3390/genes13091641

**Published:** 2022-09-13

**Authors:** Felipe Avila, Shayne S. Hughes, K. Gary Magdesian, Maria Cecilia Torres Penedo, Rebecca R. Bellone

**Affiliations:** 1 Veterinary Genetics Laboratory, School of Veterinary Medicine, University of California—Davis, Davis, CA 95616, USA; 2 Department of Medicine and Epidemiology, School of Veterinary Medicine, University of California—Davis, Davis, CA 95616, USA; 3 Department of Population Health and Reproduction, School of Veterinary Medicine, University of California—Davis, Davis, CA 95616, USA

**Keywords:** pigmentation, white spotting, genetic testing, equine

## Abstract

Since domestication, horses have been selectively bred for various coat colors and white spotting patterns. To investigate breed distribution, allele frequencies, and potential lethal variants for recommendations on genetic testing, 29 variants within 14 genes were investigated in 11,281 horses from 28 breeds. The recessive chestnut *e^a^* allele in melanocortin 1 receptor (*MC1R*) (p.D84N) was identified in four breeds: Knabstrupper, Paint Horse, Percheron, and Quarter Horse. After filtering for relatedness, *e^a^* allele frequency in Knabstruppers was estimated at 0.035, thus illustrating the importance of testing for mate selection for base coat color. The Rocky Mountain Horse breed had the highest allele frequency for two of the dilution variants under investigation (*Z*_a.f._ = 0.32 and *Ch*_a.f._ = 0.026); marker-assisted selection in this breed could aid in the production of horses with desirable dilute coats with less severe ocular anomalies caused by the silver (*Z*) allele. With regard to white patterning, nine horses homozygous for the paired box 3 (*PAX3*) splashed white 2 (*SW2*) allele (p.C70Y) and six horses homozygous for the KIT proto-oncogene, receptor tyrosine kinase (*KIT*) sabino 1 (*SB1*) allele (ECA3g.79544206A>T) were identified, thus determining they are rare and confirming that homozygosity for *SW2* is not embryonic lethal. The *KIT* dominant white 20 (*W20*) allele (p.R682H) was identified in all but three breeds: Arabian (n = 151), Icelandic Horse (n = 66), and Norwegian Fjord Horse (n = 90). The role of *W20* in pigmentation across breeds is not well understood; given the different selection regimes of the breeds investigated, these data provide justification for further evaluating the functional role of this allele in pigmentation. Here, we present the largest dataset reported for coat color variants in horses to date, and these data highlight the importance of breed-specific studies to inform on the proper use of marker-assisted selection and to develop hypotheses related to pigmentation for further testing in horses.

## 1. Introduction

Since domestication, horses have been selectively bred for a variety of pigmentation phenotypes [1]. Coat color is an important economic trait, and in some cases, it is a breed-defining phenotype. Coat color is also relevant from a health perspective, as some equine pigmentation variants have been connected to genetic disorders. One of the first variants discovered in the horse was a recessive mutation in the melanocortin 1 receptor (*MC1R*), which causes the chestnut phenotype, characterized by red-pigmented hair in the body and points (mane, tail, lower legs, and ear rims) [2]. Since then, over 60 variants contributing to equine pigmentation have been identified [3].

Coat color phenotypes can be divided into three categories: base coat color, pigmentation dilution, and white patterning. The base coat color for horses is typically characterized as one of three colors: black, bay, or chestnut. Variants in *MC1R* (also called the Extension or Red Factor locus) and its antagonist, agouti signaling protein (*ASIP*), are associated with the type and location (body and points) of melanin produced—eumelanin (black/brown pigment) or pheomelanin (red/yellow pigment). In the absence of other modifiers, horses that are homozygous for the recessive *ASIP* allele (denoted as *a*)—an 11bp deletion in exon 2 (ENSECAT00000004772.2:c.191_201del)—have an all-black phenotype (black body and black points). Signaling through MC1R allows for pigment switching; the dominant *ASIP* allele (*A*) causes eumelanin to be restricted to the points, and when in combination with the dominant *MCIR* (*E*) allele, pheomelanin is produced in the body and results in a bay phenotype (red body with black points) [4]. Individuals homozygous for the loss of function recessive *MC1R* variant (denoted as *e*, p.S83F) will only produce pheomelanin and will be a shade of chestnut (red body and red points), regardless of the *ASIP* genotype [5]. A second loss-of-function *MC1R* mutation, also associated with chestnut base color, was discovered in 2000 and termed *e^a^* (p.D84N) [6]. To date, this allele has only been detected in Black Forest, Hungarian Coldblood, and Haflinger breeds [6,7].

Variants in six genes—major facilitator superfamily domain containing 12 (*MFSD12*), myosin VA (*MYO5A*), premelanosome protein (*PMEL*), solute carrier family 36 member 1 (*SLC36A1*), solute carrier family 45 member 2 (*SLC45A2*), and T-box transcription factor 3 (*TBX3*)—have been shown to contribute to a reduction in the amount of melanin produced, resulting in a diluted coat color [8,9,10,11,12,13,14,15]. Some of these variants only affect eumelanin or pheomelanin, while others are known to reduce the amount of both pigments. The variant in *MFSD12* (p.Asp201fs), causing the mushroom phenotype in Shetland Ponies, for example, reduces pheomelanin to create a dilute sepia coat [14]. The silver phenotype, on the other hand, is caused by a missense mutation in the *PMEL* gene (p.R625C) and is hypothesized to affect the deposition of eumelanin only, thus diluting the amount of pigment in black horses and diluting the black mane of bay horses [8,9]. The breed distribution for the aforementioned dilution alleles has not previously been reported.

White patterning variants are the most numerous among those identified to impact pigmentation in horses, with 49 mutations in seven genes—endothelin receptor type B (*EDNRB*), KIT proto-oncogene, receptor tyrosine kinase (*KIT*), melanocyte inducing transcription factor (*MITF*), paired box 3 (*PAX3*), ring finger and WD repeat domain 3 (*RFWD3*), syntaxin 17 (*STX17*), and transient receptor potential cation channel subfamily M member 1 (*TRPM1*)—identified to date [3,16,17,18,19,20,21,22]. Of those, 35 were identified in or thought to regulate the *KIT* gene (dominant white 1–16, 17a–b, 18–28, 30–33, sabino 1, and tobiano) [3]. White patterns occur on any base color and/or dilution background. The phenotypic expression of white patterning variants is extremely variable, ranging from minimal white patches on the body to an all-white horse [3,20]. Genetic tests are available and routinely performed for 16 of these variants (lethal white overo, sabino 1, tobiano, *W4*, *W5*, *W10*, *W20*, *W22*, *SW1*–*SW6*, gray, leopard complex spotting, and Appaloosa Pattern-1) to assist breeders in producing horses with desired white patterns while avoiding potential health concerns [3]. Studies suggest that homozygosity for some white patterning variants is embryonic lethal, and some white patterning mutations are thought to be restricted to a specific breed, lineage, or a single individual, although more work is needed in both areas. For example, the *KIT* dominant white 5 (*W5*) variant (p.Thr732GlnfsX9) is thought to be restricted to one Thoroughbred family [21] and proposed to be homozygous lethal. Similarly, the recently discovered de novo splashed white 6 (*SW6*) variant in an American Paint Horse stallion, a 8.7 kb deletion in *MITF* (NC_009159.3:g.21551060-21559770del) that causes extensive white markings, is also hypothesized to be homozygous embryonic lethal [20]. Therefore, further investigation into potential homozygous lethal variants and a more comprehensive characterization of their breed distribution are needed.

Genetic testing for coat color variants is increasingly being utilized by horse breeders; thus, proper use of test results is important to ensure appropriate mate selection to produce the desired phenotypes. Furthermore, several studies have demonstrated the pleiotropic effects of pigmentation variants that lead to congenital disorders in horses, ranging from deafness [23] to ocular issues [24] to lethality [25]. In some cases, only homozygous individuals are affected, as in the case of lethal white overo [25], congenital stationary night blindness [26], and potentially other embryonic lethal white variants mentioned above. In other cases, homozygotes are more severely affected than heterozygotes; for example, multiple congenital ocular anomalies (MCOA) are associated with the same mutation in the *PMEL* gene that causes silver coat dilution [8,9]. Homozygotes have a more severe ocular disease that includes cataracts, iris stromal hypoplasia, abnormal pectinate ligaments, megaloglobus, and iridociliary cysts, whereas heterozygotes typically present only with iridociliary cysts [8,9]. Therefore, genetic testing can be a powerful selection tool for producing desirable traits while limiting pleiotropic anomalies in breeds in which such alleles are found. An understanding of breed-specific allelic and genotypic distribution can inform the proper utilization and interpretation of the results for this purpose. Here, we aim to further evaluate potential homozygous lethal variants and investigate the breed distribution and allele frequencies across 28 breeds of variants in 14 genes known to contribute to equine pigmentation phenotypes.

## 2. Materials and Methods

Genotypes for 29 equine coat color variants routinely tested at the University of California–Davis Veterinary Genetics Laboratory (VGL) were retrieved for 11,281 horse samples from 28 different breeds, for which data from at least 30 individuals were available. Data were restricted to breeds with at least 30 individuals for the accurate estimation of allele frequencies [27]. The number of individuals per breed ranged from 36 (Hanoverian) to 5519 (Quarter Horse) (mean = 403 horses/breed; median = 96 horses/breed; Supplementary Table S1). Genotyping data included five alleles contributing to base coat color: agouti (*A* or *a*) and extension (*E*, *e^a^*, *e*); eight alleles that cause dilution of pigment or primitive marks: champagne (*Ch*), cream (*Cr*), dun (*D*), non-dun1 (*nd1*), non-dun2 (*nd2*), mushroom (*Mu*), pearl (*Prl*), and silver (*Z*); and 16 white patterning alleles: appaloosa pattern-1 (*PATN1*), dominant white 5, 10, 20, 22 (*W5*, *W10*, *W20*, *W22*), gray (*G*), leopard Complex (*LP*), lethal white overo (*O*), splashed white 1–6 (*SW1*–*SW6*), sabino 1 (*SB1*), and tobiano (*TO*) [3,28,29]. The details of the genetic variants tested and their respective nomenclatures can be found in Supplementary Table S2.

To determine allelic and genotypic distributions across breeds, all variants were first evaluated without considering relatedness among individuals within breeds. However, to estimate breed-specific allele frequencies (a.f.) of variants in this study, the initial dataset was filtered for within-breed relatedness by removing first-degree relatives based on the date of sample submission; i.e., genotypes for the oldest banked sample were included in the study, and all of its first-degree relatives subsequently tested were removed from the dataset. The filtered cohort comprised 6,878 individuals representing 28 breeds, with the number of horses per breed ranging from 31 (Hanoverian) to 3,193 (Quarter Horse) (mean = 246 horses/breed; median = 78 horses/breed; Supplementary Table S1). Allele frequencies were calculated using the GenAlex 6.502 add-in [30,31] for Microsoft Excel 2016 (Microsoft Corporation, Redmond, Washington, United States).

## 3. Results

### 3.1. Base Color Loci

The recessive alleles contributing to the base coat colors chestnut (*e*, in *MC1R*) and black (*a*, in *ASIP*) were identified in all 28 breeds included in this study, with average estimated frequencies of 0.44 and 0.58 across breeds, respectively (Supplementary Tables S3 and S4). The frequency of the *e* allele ranged from 0.76 in Pony of the Americas to 0.12 in Percheron. Estimated frequencies for the *a* allele were highest in Percheron (a.f. = 0.99), Gypsy Vanner (a.f. = 0.92), Rocky Mountain Horse (a.f. = 0.87), Tennessee Walking Horse (a.f. = 0.86), and Gypsy Cob (a.f. = 0.82), and lowest in the Thoroughbred (a.f. = 0.24) and Norwegian Fjord Horse (a.f. = 0.24). The *e^a^* allele in *MC1R* was observed in four breeds: Knabstrupper (5 *E*/*e^a^* and 1 *e*/*e^a^*), Paint Horse (1 *e*/*e^a^*), Percheron (1 *E*/*e^a^*), and Quarter Horse (1 *E*/*e^a^* and 1 *e*/*e^a^*) (Table 1). After filtering for relatedness, the *e^a^* allele frequency in the Knabstrupper was estimated at 0.035 (Supplementary Table S4).

### 3.2. Dilution Loci

The champagne allele (*Ch*) was identified in 8 breeds: Gypsy Cob (a.f. = 0.0080), Miniature Horse (a.f. = 0.017), Missouri Fox Trotter (a.f. = 0.054), Paint Horse (a.f. = 0.0070), Quarter Horse (a.f. = 0.0080), Rocky Mountain Horse (a.f. = 0.026), Shetland Pony (a.f. = 0.0020), and Tennessee Walking Horse (a.f. = 0.0080). The cream allele (*Cr*) was observed in 25 of the 28 breeds in this study and was not found in the Hanoverian, Knabstrupper, and Percheron breeds. Allele frequencies for this variant ranged from 0.42 in Lusitano to 0.013 in Standardbreds. Conversely, another dilution variant in the *SLC45A2* gene, pearl (*Prl*; p.A329T) [12], was only identified in 7 breeds: Andalusian (a.f. = 0.12), Gypsy Cob (a.f. = 0.017), Gypsy Vanner (a.f. = 0.015), Lusitano (a.f. = 0.064), Paint Horse (a.f. = 0.0070), Pura Raza Española (a.f. = 0.11), and Quarter Horse (a.f. = 0.00031).

The dominant *D* allele causing the dun dilution phenotype with primitive marks was not detected in 9 breeds analyzed: Arabian, Dutch Warmblood, Gypsy Vanner, Hanoverian, Knabstrupper, Oldenburg, Percheron, Standardbred, and Thoroughbred. In breeds in which this allele was detected, it was estimated to be fixed (a.f. = 1.0) in the Norwegian Fjord Horse, whereas the lowest allele frequency was estimated for the Tennessee Walking Horse (a.f. = 0.0060). The *nd1* (non-dun 1) allele, which causes primitive marks without diluting the coat, was identified in all breeds except for Gypsy Vanner and Norwegian Fjord Horse, and its frequency ranged from 0.79 in Pura Raza Española to 0.017 in Gypsy Cob. Similarly, the *nd2* (non-dun 2) allele (no dun dilution and no primitive marks) was found in all breeds, except for the Norwegian Fjord Horse. Its allele frequencies were relatively high across all breeds investigated, ranging from 1.0 in Gypsy Vanner to 0.20 in Pura Raza Española.

The mushroom (*Mu*) allele, while only tested in Icelandic Horse, Miniature Horse, Quarter Horse, and Shetland Pony, was detected only in Shetland Ponies, with an allele frequency after filtering for relatedness of 0.23. Finally, the silver (Z) allele was found in 15 breeds, with the highest allele frequency estimated for Rocky Mountain Horse (a.f. = 0.32) and the lowest in Quarter Horse (a.f. = 0.0011). Unfiltered genotype counts and allele frequencies for the filtered dataset can be found in Supplementary Tables S3 and S4, respectively.

### 3.3. White Patterning Loci

Homozygotes for three of the four *KIT* dominant white variants investigated (*W5*, *W10*, *W22*) were not detected in our dataset (Table 2). Compound heterozygotes were detected only for *W20* (one *W10*/*W20* and three *W20*/*W22*). The dominant white 20 (*W20*) variant was found to have a widespread distribution, detected in 25/28 breeds included in this study, with a total of 340 *W20* homozygotes from 21 breeds (Table 2). The allele frequency for this variant was lowest in the Rocky Mountain Horse (a.f. = 0.0090) and highest in the Gyspy Cob (a.f. = 0.33) and Thoroughbred (a.f. = 0.34). The *W20* allele was not detected in Arabians (n = 180), Icelandic Horses (n = 73), and Norwegian Fjord Horses (n = 121). The dominant white 5 (*W5*) in *KIT* was only identified in two related heterozygous Thoroughbreds, with a population filtered allele frequency of 0.0070 (Supplementary Tables S2 and S3). Conversely, the *W10* variant was found in two Paint Horses (*N*/*W10*) and two Quarter Horses (one *N*/*W10* and one *W10*/*W20*), whereas the *W22* mutation was identified in one Oldenburg (a.f. = 0.011), 4 Paint Horses (a.f. = 0.00096), 6 Quarter Horses (a.f. = 0.00054), and 2 Thoroughbreds (a.f. = 0.014). All horses with *W22* can be traced back to the ancestor, originally described as the founder [22].

**Figure 1:**
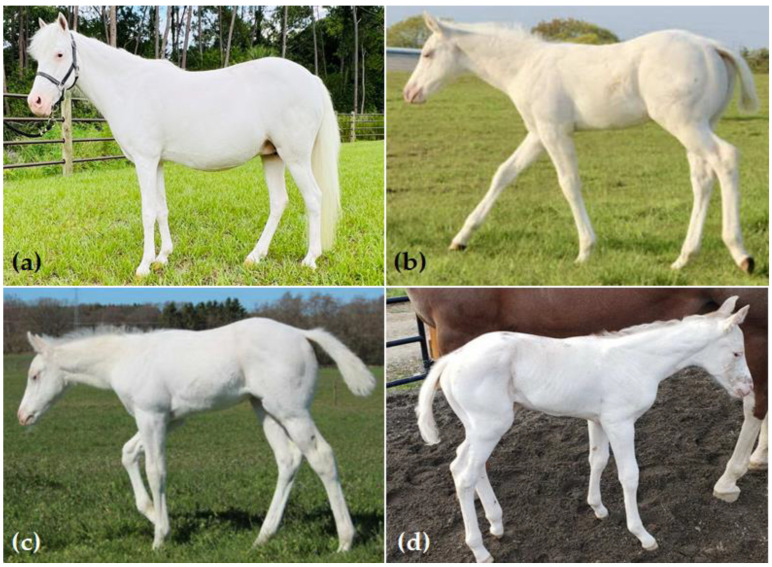
Four horses homozygous for the *SB1* white patterning allele (*SB1*/*SB1*). (**a**) Shetland Pony with chestnut base coat and silver dilution (*e/e a/a Z/N*) with no other tested dilution or white patterning alleles detected; (**b**) Paint Horse with chestnut base coat (*e/e A/A*); (**c**) Paint Horse with bay base coat (*E/e A/A*); (**d**) Paint Horse with bay base coat (*E/e A/A*); (**b**–**d**) No other tested dilution or white patterning alleles were detected in these horses.

Allele frequencies were estimated across breeds for two additional *KIT* variants: sabino 1 (*SB1*) and tobiano (TO). *SB1* was identified in 171 horses from 10 breeds, namely Gypsy Cob, Gypsy Vanner, Miniature Horse, Missouri Fox Trotter, Mustang, Paint Horse, Pony of the Americas, Quarter Horse, Shetland Pony, and Tennessee Walking Horse (Supplementary Table S3). Allele frequencies for *SB1* were the highest in Tennessee Walking Horse (a.f. = 0.083) and the lowest in Quarter Horse (a.f. = 0.0010) (Supplementary Table S4). *SB1* is reported to follow an incompletely dominant inheritance pattern, with homozygotes displaying a more pronounced phenotype [32]. In this large dataset, only six individuals were homozygous for this variant (4 Paint Horses, 1 Shetland Pony, and 1 Tennessee Walking Horse), and photographic records were available for four of these (Figure 1), all of which displayed an all-white phenotype without any other known white spotting variants (Table 3).

Tobiano (*TO*) [33] was more widely distributed and was identified in 787 horses from 15 breeds, with allele frequencies ranging from 0.22 in Shetland Ponies to 0.0033 in Arabian Horses (Supplementary Table S4). A total of 179 *TO/TO* homozygotes were identified in 9 breeds: Appaloosa, Gypsy Cob, Gypsy Vanner, Icelandic Horse, Miniature Horse, Missouri Fox Trotter, Paint Horse, Shetland Pony, and Tennessee Walking Horse (Supplementary Table S3). Investigating the distribution of horses with multiple variants at the *KIT* locus identified 140 horses with a combination of *W20*, *SB1*, and/or *TO* (Table 4). The majority of individuals with both *TO* and *W20* alleles were found in Paint Horse (n = 59) and Gypsy Cob (n = 12). There were 12 additional Paint Horses found to have both *SB1* and *W20* (Table 4).

The gray (*G*) variant had a wide distribution, as it was detected in all breeds studied, except for Knabstrupper (n = 91) and Norwegian Fjord Horse (n = 121). The highest frequency for this allele was observed in Connemara Pony (a.f. = 0.31), whereas the lowest was found in Icelandic and Morgan Horses (a.f. = 0.0080). The lethal white overo (*O*) variant was identified in 422 samples across 11 breeds: Andalusian, Appaloosa, Arabian, Miniature Horse, Missouri Fox Trotter, Mustang, Paint Horse, Quarter Horse, Shetland Pony, Tennessee Walking Horse, and Thoroughbred. Estimated allele frequencies after relatedness filtering ranged from 0.0020 in the Quarter Horse and 0.0025 in Appaloosa to 0.030 in the Thoroughbred and 0.10 in the Paint Horse.

The leopard complex spotting (*LP*) allele of *TRPM1* was identified in 460 horses from 13 breeds: 1 Andalusian, 192 Appaloosas, 8 Gypsy Cobs, 1 Gypsy Vanner, 75 Knabstruppers, 120 Miniature Horses, 1 Mustang, 1 Paint Horse, 48 Pony of the Americas, 2 Quarter Horses, 7 Shetland Ponies, 2 Tennessee Walking Horses, and 1 Welsh Pony. The highest *LP* allele frequencies, unsurprisingly, were detected in those breeds where the pattern is a breed-defining phenotype: Appaloosa (a.f. = 0.60), Pony of the Americas (a.f. = 0.64), and Knabstrupper (a.f. = 0.58) (Supplementary Table S4). The appaloosa pattern-1 (*PATN1*) variant, a modifier of *LP* that controls for a high amount of white patterning when inherited with *LP* [34], was identified in all 13 breeds where *LP* was found, as well as Connemara Pony (a.f. = 0.066), Missouri Fox Trotter (a.f. = 0.011), and Rocky Mountain Horse (a.f. = 0.0040) (Supplementary Table S4). Moreover, the estimated allele frequency of *PATN1* was higher than that of *LP* in three breeds: Gypsy Vanner, Shetland Pony, and Welsh Pony (Table 5).

The splashed white phenotype is controlled by at least six different mutations in two genes. Consistent with previous studies [35], the *MITF SW1* allele was the most widespread of these variants, identified in 599 horses from 13 breeds with homozygotes (*SW1/SW1*, n = 49) identified in six of these: Appaloosa (n = 1), Icelandic Horse (n = 5), Miniature Horse (n = 5), Morgan Horse (n = 4), Paint Horse (n = 11), and Quarter Horse (n = 23). Allele frequencies were highest in the Miniature Horse (a.f. = 0.10) and lowest in the Shetland Pony (a.f. = 0.0070) (Table 6).

**Figure 2:**
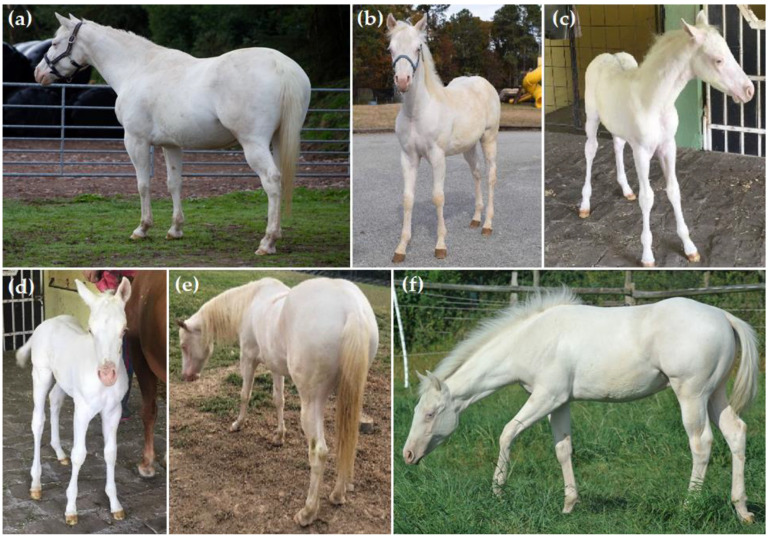
Six Quarter Horses homozygous for the *SW2* white patterning allele (*SW2/SW2*). (**a**) Bay base coat with cream dilution, known as buckskin, (*E/e A/A Cr/N*) and having other white pattern alleles (*SW1/N W20/N*); (**b**) Bay base coat (*E/e A/A*) and other white patterning alleles (*SW1/N W20/N*); (**c**) Bay base coat (*E/e A/A*) and *SW1/N W20/N*; (**d**) Bay base coat (*E/e A/a*) and *SW1/N W20/N*; (**e**) chestnut base coat with cream allele, known as Palomino (*e/e A/A Cr/N*), with no other dilution or white patterning alleles detected; (**f**) Bay base coat (*E/E A/a*) and homozygous *W20/W20*.

The *PAX3* splashed white 2 (*SW2*) variant was found in 274 horses from only two breeds: Paint Horse (n = 66) and Quarter Horse (n = 208) (Table 6). A total of three Paint Horses and six Quarter Horses were identified as homozygous for the *SW2* variant. Photographic records were available for six Quarter Horses, with each displaying an all-white phenotype, although five of these also had other known white spotting alleles (Figure 2).

After removing related individuals, the frequency was low in both breeds (a.f. = 0.0078 for Paint Horse and a.f. = 0.0083 for Quarter Horse, Table 6). No homozygotes for *SW3*, *SW4*, *SW5*, or *SW6* were identified in this dataset. The *PAX3* splashed white 4 (*SW4*) allele was not identified in any horses in this study. The *MITF SW3* allele was identified in five Paint Horses and seven Quarter Horses, with a very low frequency after filtering for relatedness in each breed (a.f. ≤ 0.0010). Finally, *SW5* and *SW6* were restricted to Paint Horses (with one exception for *SW6*, a double registered AQHA/APHA Quarter Horse), with allele frequencies of 0.0020 and 0.00056, respectively. One Paint Horse *SW1/SW6* compound heterozygote was identified. The only other combinations of splashed white variants identified in the dataset involved *SW1* and *SW2* in Paint Horses and Quarter Horses (Table 6, Figure 2).

## 4. Discussion

The base coat color variants for chestnut (*e*, in the *MC1R* gene) and black (*a*, located in *ASIP*) were identified in all 28 breeds analyzed in this study (Supplementary Table S3). The *a* allele was found to be nearly fixed in the Percheron (a.f. = 0.99), a breed highly selected for black and gray coat phenotypes. The *MC1R* allele *e^a^* [6], previously reported in chestnut horses in Black Forest, Hungarian Coldblood, and Haflinger breeds [6,7], was identified for the first time here in Paint Horse, Percheron, and Quarter Horse. Moreover, since several Knabstruppers with the *e^a^* allele were detected in this study, we were able to report, for the first time, an estimated allele frequency for *e^a^* in this breed (a.f. = 0.035). Knabstruppers are selectively bred for leopard complex spotting patterns, and a particular base color is often desired. Therefore, genotyping that allows for the accurate detection of horses with the *e^a^* allele is important for marker-assisted selection. No homozygous *e^a^/e^a^* individuals were identified in this study, although they have been reported in the Black Forest breed [6].

Dilution alleles, on the other hand, were found to be restricted in their breed distribution and show relatively low allele frequencies, with the exception of *Cr*. Nonetheless, some interesting observations can be made. Concerning the *Ch* allele, it was found at a low frequency in eight breeds, but interestingly, the breed in which the variant was first discovered, Tennessee Walking Horse [13], is among those breeds with the lowest estimated frequency (a.f. = 0.0080). Conversely, the breed with the highest frequency, Rocky Mountain Horse (a.f. = 0.026), has not previously been reported to have this variant. No known adverse health effects have been reported to be associated with champagne dilution. Additionally, given the high allele frequency of the silver variant in Rocky Mountain Horses (a.f. = 0.32) and its association with MCOA, the use of marker-assisted selection for silver heterozygosity and champagne may yield breed-desirable coat color dilutions with fewer potential ocular issues. Horses homozygous for the *PMEL* silver mutation (*Z/Z*) are reported to have a more severe form of disease that can impair vison or cause blindness, whereas heterozygotes (*Z/N*) are often reported to have a cyst-only phenotype. The *Z* allele was identified in 15 other breeds, and in addition to the Rocky Mountain Horse, it was previously reported in Miniature Horse, Missouri Fox Trotter, Icelandic Horse, Shetland Pony, and Morgan Horse. Here, we also identified the *Z* allele in Mustang, Tennessee Walking Horse, Gypsy Vanner, Welsh Pony, Dutch Warmblood, Gypsy Cob, Pony of the Americas, Lusitano, Quarter Horse, and Paint Horse for the first time (Supplementary Table S3). Given the known pleiotropic effects of this variant, breeds in which it occurs are advised to utilize genetic testing for mate selection, as well as a tool to identify horses who should be examined by a veterinary ophthalmologist for MCOA.

In the case of *Prl*, while identified at a relatively low frequency in seven breeds, the highest frequencies were found in Iberian horses. Similar allele frequencies were estimated for the Andalusian and closely related Pura Raza Española breed (a.f. = 0.12 and 0.11, respectively), as well as the Lusitano (a.f. = 0.064) (Supplementary Table S3). To our knowledge, this is the first time that this variant has been reported in Lusitano and Pura Raza Española. Given that the highest frequency of *Prl* is in Iberian breeds, it is possible that it is undergoing positive selection because of the recent use of genetic testing and/or changes in studbook rules in the Pura Raza Española starting in 2002, which allowed the registration of additional color phenotypes other than black, bay, and gray. The *Prl* allele is recessive and is known to dilute only the base color when homozygous or when combined with the cream (*Cr*) allele. Iberian breeds were also among those with the highest allele frequencies for *Cr*, with the highest being in Lusitano (a.f. = 0.42), which again indicates selection for coat color dilution in these breeds. Given the relatively high frequency of *Cr* and *Prl* in these breeds, genetic testing for these variants can easily assist in the consistent production of these desirable phenotypes.

The *Mu* allele was originally reported in Shetland Ponies with an allele frequency of 0.12 (n = 177), and in that study, it was also identified in Miniature Horses at a low frequency (a.f. = 0.020; n = 129) [14]. In this study, the *Mu* allele was only identified in Shetland Pony, as genotyping data were only available for two Miniature Horses. Here, the *Mu* allele frequency in Shetland Pony after filtering for relatedness was estimated at 0.23 (n = 173). This was nearly double that previously reported by us [14]; however, this is unlikely to reflect a true increase in population frequency despite being a favorable trait in the breed. Since the discovery was reported in 2019, the *Mu* allele frequency estimated herein more likely represents a sampling bias for horses owned and tested by breeders who breed for this trait. Testing a larger randomized cohort is needed to investigate this further.

Concerning the dun dilution characterized by lightening of body hair and the presence of primitive markings, including a darker dorsal stripe, leg and/or shoulder stripes, and dark marks known as cobwebbing on the forehead, the Norwegian Fjord Horse was found to be fixed for the wild-type *D* allele (a.f. = 1.0, n = 121). To the best of our knowledge, this is the first documented molecular investigation of the frequency of this variant in the breed. However, it has been previously suggested that this allele was fixed, or nearly so, given the breed-defining dun phenotype characteristic of Norwegian Fjords [5]. The *nd1* allele, which leads to the expression of primitive markings without dilution of the coat, was estimated to be at high frequency in Iberian horse breeds as well as in the Arabian: Pura Raza Española (a.f. = 0.79), Andalusian (a.f. = 0.78), Lusitano (a.f. = 0.57), and Arabian (a.f. = 0.68). These findings likely reflect the common ancestry of Iberian horse breeds, which were developed using Arabians brought to the Iberian Peninsula during the Muslim invasions of the 8th century. Given the frequency of *nd1* in Iberian and Arabian breeds, investigating whether this variant contributes to the darker shade or countershading is worthy of further exploration and may help to better understand why the *nd1* allele appears to be under positive selection.

Dominant white phenotypes and their associated alleles were originally named as such because they were believed to be lethal when homozygous. Consistent with this, no homozygotes for *W5*, *W10*, or *W22* were identified in this study. However, due to their low allele frequencies and limited breed distribution (Supplementary Table S4), the probability of identifying homozygotes in the population is extremely low, regardless of lethality. Consistent with previous work [22,36], homozygotes were identified for the *W20* allele in the 21 breeds included in this study. These breeds represent a variety of coat color selection preferences, ranging from horses with breed-defining phenotypes that include white patterning, such as the Paint Horse (a.f. = 0.21) and the Appaloosa (a.f. = 0.18), to breeds that prohibit excessive white markings, such as the Hanoverian (a.f. = 0.24). The role of *W20* in pigmentation across breeds is not well understood. Previous research supports that *W20* (p.Arg682His) leads only to a minor reduction in *KIT* function [36]. However, it has been documented that *W20,* in combination with other dominant white alleles, causes higher amounts of white patterning [22,36]. Based on our findings, compound heterozygotes for *W10/W20* (one Quarter Horse) and *W20/W22* (three Quarter Horses) are rare (Table 2). It is important to note here that the *W22* variant occurs on the sequence background of *W20* [22]. A detailed investigation of the phenotypic effects of *W20* in combination with other white-patterning alleles has not yet been performed. Here, we identified 93 horses from nine breeds (Appaloosa, Gypsy Cob, Gypsy Vanner, Miniature Horse, Missouri Fox Trotter, Paint Horse, Shetland Pony, Tennessee Walking Horse, and Welsh Pony) with both the *TO* allele and *W20* (Table 4). Thirty-three horses across nine breeds (Gypsy Cob, Gypsy Vanner, Miniature Horse, Missouri Fox Trotter, Mustang, Paint Horse, Quarter Horse, Shetland Pony, and Tennessee Walking Horse) had *SB1* and *W20* (Table 4, Supplementary Table S3). Photographic records were not available for most of the horses in this study but given the occurrence of these combinations of *KIT* mutations across several breeds, a formal investigation of their potential additive effects on white pattering is warranted. A recent study of American Paint Horses showed that, in the absence of any other known white patterning variants, *W20* was associated with white spotting phenotypes defined by the American Paint Horse Association (APHA) [37]. However, the possibility that undiscovered variants contribute to the phenotypes, to our knowledge, has not yet been explored. The widespread across-breed distribution of *W20*, combined with high estimated allele frequencies in breeds not typically selected for white patterning in this study, provides further justification for evaluating the functional role of this allele in pigmentation across breeds. This knowledge will be essential in order to develop breed-specific recommendations for utilizing *W20* genotypes to maximize breeding potential for desired coat color phenotypes.

Some of the splashed white variants were predicted to be homozygous lethal, and in this study, we only identified homozygotes for *SW1* (n = 49) and *SW2* (n = 9) (Table 6). Previous studies have shown that *SW1* homozygotes are viable and have an all-white or nearly all-white phenotype [35]. Two homozygous *SW2/SW2* horses have been reported in the literature, with only one having a documented phenotype [37]. Having confirmed genotypes of nine *SW2* homozygotes across two breeds (Paint Horse and Quarter Horse)—the most reported to date—and in evaluating photographic records of six of these horses, we confirm that this genotype is not lethal. All six *SW2/SW2* horses had an all-white phenotype, but five of the six also had other white spotting variants (Figure 2). Thus, further evaluation of *SW2* homozygotes without other known white pattern alleles will substantiate the hypothesis that this genotype alone produces an all-white phenotype. One owner reported that their *SW2/SW2* horse was deaf, but this remains to be clinically evaluated in this individual, as well as in other *SW2* homozygotes.

Another rare non-lethal white patterning variant is *SB1*. The sabino 1 phenotype is described as extensive face and leg markings, along with white in the belly and roaning in the flanks [32]. The causal *KIT* variant was first reported in the Tennessee Walking Horse, American Miniature Horse, Paint Horse, Azteca, Missouri Foxtrotter, Shetland Pony, and Spanish Mustang [32]. A subsequent study investigated this variant in 899 horses across eight breeds and identified *SB1* in three additional breeds (Haflinger, Noriker, and Lippizan), but no homozygotes were observed [38]. Here, we report the occurrence of the *SB1* allele in Gypsy Cob, Gypsy Vanner, Pony of the Americas, and Quarter Horse for the first time. Furthermore, we identified the largest number of *SB1* homozygotes to date, with six *SB1/SB1* individuals across three breeds (Paint Horse, Shetland Pony, and Tennessee Walking Horse). Photographic records were available for four of these horses, and consistent with a previous report [37], they have an all-white phenotype (Figure 1). Given that *SB1* and *SW2* homozygotes are viable but rare, genetic testing can aid in the identification of these rare mates so that 100% of offspring have a white patterning allele.

Concerning leopard complex spotting, we report the first molecular detection and allele frequency of *LP* and *PATN1* in breeds outside of those used to discover mutations [26,34] (Table 5). Furthermore, in those breeds that are selected for *LP*, this is the first time that the frequency of the modifying locus *PATN1* has been concurrently evaluated. Consistent with photographic records and breeding schemes, the allele frequency for *PATN1* is higher in the Knabstrupper than in Appaloosa, Pony of the Americas, or Miniature Horse. Knowing the frequency in these breeds can help guide selection strategies away from homozygosity for *LP*, which causes congenital stationary night blindness (CSNB) and an increased risk of insidious uveitis (ERU) [26,39,40]. Horses homozygous for *LP* are night blind due to premature polyadenylation of *TRPM1,* which in turn is predicted to lead to no functional TRPM1 protein in the bipolar cells of the retina. In the case of ERU, horses homozygous for *LP* are at a higher risk of this disease, but the biological mechanism for this increased risk remains unknown [39,40]. Genotyping for LP in breeds where the allele is present can help breeders select for *LP* heterozygotes, which, when in combination with the *PATN1* variant, display the desirable leopard pattern without being afflicted with night-blindness [26]. Interestingly, *PATN1* was identified in three breeds where no *LP* horses were detected (Connemara Pony, Missouri Fox Trotter, and Rocky Mountain Horse); moreover, *PATN1* allele frequency was estimated to be higher than that of *LP* in three breeds: Gypsy Vanner, Shetland Pony, and Welsh Pony (Table 5). It is currently unknown whether *PATN1* acts as a modifier for other white patterning loci; therefore, investigating white pattern levels in these breeds where *PATN1* but not *LP* was identified, specifically in horses with other white patterning alleles, is warranted.

In conclusion, these data represent the largest study to date investigating pigmentation variants in horses across a large number of breeds. We reported estimated allele frequencies for base coat color, dilution, and white patterning genes in 28 breeds. While we restricted our dataset to breeds with 30 or more samples, a limitation of the study is that these samples were originally submitted for coat color testing. Therefore, it is possible that in those breeds with fewer individuals evaluated, estimates may be biased upwards, as noted above, for the mushroom variant. We identified the presence of several variants in breeds not previously reported. Additionally, we identified and reported nine *SW2/SW2* homozygotes, the most to date, and confirmed that this genotype is not lethal. Here, we also report the largest number of *SB1* homozygotes to date (n = 6), with photographic records for most to corroborate its effect on phenotype. Understanding breed distribution and allele frequencies can help guide recommendations on and utilization of genetic testing for marker-assisted selection. Furthermore, these findings will help guide future hypothesis-driven studies toward a better functional understanding of these pigmentation variants in and across breeds.

## Figures and Tables

**Table 1:** Observed genotype counts for the *MC1R* locus in the four breeds in which the chestnut *e^a^* allele (*MC1R*:p.D84N) was detected.

	Genotype	*E/E*	*E/e*	*E/e^a^*	*e/e*	*e/e^a^*	*e^a^/e^a^*	Individuals Tested
Breed								
Knabstrupper		29	41	5	15	1	0	91
Paint Horse		191	720	0	1164	1	0	2076
Percheron		38	13	1	0	0	0	52
Quarter Horse		1100	2484	1	1926	1	0	5512

**Table 2:** Number of individuals tested (n), number of heterozygous and homozygous, and estimated allele frequencies (a.f.) after filtering for relatedness for dominant white variants *W5*, *W10*, *W20*, and *W22*.

***W5***									
		**Genotypes-Unfiltered**							
**Breed**	**n (Unfiltered)**	***N/W5***		***W5/W5***		***W5/W20***		**n (Filtered)**	**a.f. (Filtered)**
Thoroughbred	69	2		0		0		67	0.0075
Total	69	2		0		0		-	-
***W10***									
		**Genotypes-Unfiltered**							
**Breed**	**n (Unfiltered)**	***N/W10***		***W10/W10***		**W10/W20**		**n (Filtered)**	**a.f. (Filtered)**
Quarter Horse	5517	1		0		1		3187	0.00031
Paint Horse	2077	2		0		0		1018	0
Total	7594	3		0		1		-	-
***W20***									
		**Genotypes-Unfiltered**							
**Breed**	**n (Unfiltered)**	***N/W20***			***W20/W20***			**n (Filtered)**	**a.f. (Filtered)**
Andalusian	195	5			0			132	0.011
Appaloosa	221	57			11			198	0.18
Arabian	180	0			0			151	0
Connemara Pony	89	19			1			76	0.12
Dutch Warmblood	37	13			3			34	0.26
Gypsy Cob	84	43			7			60	0.33
Gypsy Vanner	109	40			9			67	0.27
Hanoverian	36	11			3			31	0.24
Icelandic Horse	73	0			0			66	0
Knabstrupper	90	25			4			70	0.18
Lusitano	129	13			3			102	0.049
Miniature Horse	415	31			1			295	0.046
Missouri Fox Trotter	64	9			2			46	0.12
Morgan Horse	464	73			9			265	0.092
Mustang	44	12			2			40	0.20
Norwegian Fjord Horse	121	0			0			90	0
Oldenburg	46	18			3			34	0.26
Paint Horse	2077	661			128			1018	0.21
Percheron	52	2			0			37	0.027
Pony of the Americas	58	17			4			43	0.21
Pura Raza Espanola	100	4			0			80	0.025
Quarter Horse	5517	1234			127			3187	0.14
Rocky Mountain Horse	180	3			0			115	0.0087
Shetland Pony	352	7			1			260	0.013
Standardbred	39	2			0			39	0.026
Tennessee Walking Horse	340	40			1			181	0.072
Thoroughbred	69	22			13			67	0.34
Welsh Pony	86	33			6			79	0.26
Total	11,267	2394			338			-	-
***W22***									
		**Genotypes-Unfiltered**							
**Breed**	**n (Unfiltered)**	***N/W22***		***W22/W22***		***W20/W22***		**n (Filtered)**	**a.f. (Filtered)**
Oldenburg	46	1		0		0		34	0.015
Paint Horse	2077	4		0		0		1018	0.0015
Quarter Horse	5517	3		0		3		3187	0.00047
Thoroughbred	69	2		0		0		67	0.015
Total	7709	10		0		3		-	-

**Table 3:** Individuals homozygous for *KIT* variant sabino 1 (*SB1*) with their respective breeds and genotypes for other coat color variants analyzed in this study. A dash indicates that genotyping for that locus was not performed for that individual. PH = Paint Horse; TWH = Tennessee Walking Horse; SP = Shetland Pony.

Individuals						
Coat Color Variants	PH 1	PH 2	PH 3	PH 4	TWH	SP
Sabino 1 (*SB1*)	*SB1/SB1*	*SB1/SB1*	*SB1/SB1*	*SB1/SB1*	*SB1/SB1*	*SB1/SB1*
Red Factor/Extension (*MC1R*)	*E/e*	*e/e*	*e/e*	*E/e*	*E/E*	*e/e*
Agouti (*ASIP*)	*A/A*	*A/A*	*A/A*	*A/A*	*a/a*	*a/a*
Champagne (*SLC36A1*)	*N/N*	*N/N*	*N/N*	*N/N*	*N/N*	*N/N*
Cream/Pearl (*SLC45A2*)	*N/N*	*N/N*	*N/N*	*N/N*	*N/N*	*N/N*
Dun (*TBX3*)	*nd1/nd2*	*-*	*nd2/nd2*	*nd1/nd2*	*nd1/nd2*	*nd2/nd2*
Mushroom (*MFDS12*)	*-*	*-*	*-*	*-*	*-*	*N/N*
Silver (*PMEL*)	*N/N*	*N/N*	*N/N*	*N/N*	*Z/Z*	*Z/N*
Appaloosa Pattern-1 (*RFWD3*)	*N/N*	*N/N*	*N/N*	*N/N*	*N/N*	*N/N*
*W5* (*KIT*)	*N/N*	*N/N*	*N/N*	*N/N*	*N/N*	*N/N*
*W10* (*KIT*)	*N/N*	*N/N*	*N/N*	*N/N*	*N/N*	*N/N*
*W20* (*KIT*)	*N/N*	*N/N*	*N/N*	*N/N*	*N/N*	*N/N*
*W22* (*KIT*)	*N/N*	*N/N*	*N/N*	*N/N*	*N/N*	*N/N*
Gray (*STX17*)	*N/N*	*N/N*	*N/N*	*N/N*	*N/N*	*N/N*
Leopard Complex (*TRPM1*)	*N/N*	*N/N*	*N/N*	*N/N*	*N/N*	*N/N*
Lethal White Overo (*EDNRB*)	*N/N*	*N/N*	*N/N*	*N/N*	*N/N*	*N/N*
*SW1* (*MITF*)	*N/N*	*N/N*	*N/N*	*N/N*	*N/N*	*N/N*
*SW2* (*PAX3*)	*N/N*	*N/N*	*N/N*	*N/N*	*N/N*	*N/N*
*SW3* (*MITF*)	*N/N*	*N/N*	*N/N*	*N/N*	*N/N*	*N/N*
*SW4* (*PAX3*)	*N/N*	*-*	*N/N*	*N/N*	*N/N*	*N/N*
*SW5* (*MITF*)	*N/N*	*-*	*N/N*	*N/N*	*N/N*	*N/N*
*SW6* (*MITF*)	*N/N*	*-*	*N/N*	*N/N*	*N/N*	*N/N*
Tobiano (*KIT*)	*N/N*	*N/N*	*N/N*	*N/N*	*N/N*	*N/N*

**Table 4:** Breed, number of individuals tested (n), and respective number of samples identified with multiple variants at the *KIT* locus.

Genotypes-Unfiltered				
Breed	n (Unfiltered)	*TO/W20*	*TO/SB1*	*W20/SB1*
Andalusian	195	0	0	0
Appaloosa	221	1	0	0
Gypsy Cob	84	12	2	4
Gypsy Vanner	109	6	0	2
Miniature Horse	415	5	2	1
Missouri Fox Trotter	64	2	0	2
Mustang	44	0	0	1
Paint Horse	2077	61	4	15
Quarter Horse	5517	0	0	3
Shetland Pony	352	1	0	1
Tennessee Walking Horse	340	4	6	4
Welsh Pony	85	1	0	0
Total	-	93	14	33

**Table 5:** Number of individuals (n) genotyped for *LP* and *PATN1* before and after filtering for relatedness, genotype counts per breed, and estimated allele frequencies (a.f.) for the *LP* and *PATN1* variants across 28 breeds. Bolded a.f. represents breeds in which the presence of the allele and a.f. are reported for the first time.

	Unfiltered					Filtered		
Breed	n	*LP/N*	*LP/LP*	*PATN1/N*	*PATN1/PATN1*	n	*LP* a.f.	*PATN1* a.f.
Andalusian	198	1	0	1	0	135	**0.0037**	**0.0037**
Appaloosa	221	124	68	73	0	198	0.60	0.24
Arabian	180	0	0	0	0	151	0	0
Connemara Pony	89	0	0	6	0	76	0	**0.066**
Dutch Warmblood	37	0	0	0	0	34	0	0
Gypsy Cob	84	7	1	10	0	60	**0.067**	**0.067**
Gypsy Vanner	109	1	0	16	0	67	**0.0075**	**0.10**
Hanoverian	36	0	0	0	0	31	0	0
Icelandic Horse	73	0	0	0	0	66	0	0
Knabstrupper	91	47	28	49	0	71	0.58	0.50
Lusitano	129	0	0	0	0	102	0	0
Miniature Horse	416	79	41	76	11	295	0.19	0.15
Missouri Fox Trotter	64	0	0	1	0	46	0	**0.011**
Morgan Horse	465	0	0	0	0	266	0	0
Mustang	44	1	0	1	0	40	**0.012**	**0.012**
Norwegian Fjord Horse	121	0	0	0	0	90	0	0
Oldenburg	46	0	0	0	0	34	0	0
Paint Horse	2079	1	0	9	0	1019	0.00049	**0.0024**
Percheron	52	0	0	0	0	37	0	0
Pony of the Americas	58	28	20	16	3	43	0.64	0.20
Pura Raza Espanola	100	0	0	0	0	80	0	0
Quarter Horse	5515	2	0	6	0	3191	0.00031	**0.00094**
Rocky Mountain Horse	183	0	0	1	0	117	0	**0.0043**
Shetland Pony	352	5	2	17	4	260	**0.0077**	**0.027**
Standardbred	39	0	0	0	0	39	0	0
Tennessee Walking Horse	340	2	0	0	0	181	**0.0028**	0
Thoroughbred	69	0	0	0	0	67	0	0
Welsh Pony	86	0	1	2	1	79	**0.013**	**0.025**

**Table 6:** Number of individuals tested (n), genotypes, and estimated allele frequencies (a.f.) for splashed white variants *SW1*–*SW6* after filtering for relatedness.

***SW1***					
		**Genotypes-Unfiltered**			
**Breed**	**n (Unfiltered)**	***SW1/N***	***SW1/SW1***	**n (Filtered)**	**a.f. (Filtered)**
Appaloosa	204	11	1	186	0.035
Dutch Warmblood	30	1	0	28	0.018
Gypsy Cob	82	3	0	58	0.026
Icelandic Horse	65	5	5	59	0.076
Knabstrupper	58	3	0	42	0.036
Miniature Horse	357	56	5	252	0.10
Morgan Horse	381	11	4	200	0.018
Oldenburg	44	2	0	32	0.016
Paint Horse	1872	190	11	887	0.053
Pony of the Americas	45	1	0	35	0.014
Quarter Horse	4906	257	23	2793	0.017
Shetland Pony	297	3	0	215	0.0070
Welsh Pony	67	7	0	61	0.057
Total		550	49		
***SW2***					
		**Genotypes-Unfiltered**			
**Breed**	**n (Unfiltered)**	***SW2/N***	***SW2/SW2***	**n (Filtered)**	**a.f. (Filtered)**
Paint Horse	2080	63	3	1020	0.0078
Quarter Horse	5518	202	6	3193	0.0083
Total		265	9		
***SW3***					
		**Genotypes-Unfiltered**			
**Breed**	**n (Unfiltered)**	***SW3/N***	***SW3/SW3***	**n (Filtered)**	**a.f. (Filtered)**
Paint Horse	1872	3	0	887	0.00056
Quarter Horse	4906	4	0	2793	0.00036
Total		7	0		
***SW4***					
		**Genotypes-Unfiltered**			
**Breed**	**n (Unfiltered)**	***SW4/N***	***SW4/SW4***	**n (Filtered)**	**a.f. (Filtered)**
None	None	None	None	N/A	N/A
***SW5***					
		**Genotypes-Unfiltered**			
**Breed**	**n (Unfiltered)**	***SW5/N***	***SW5/SW5***	**n (Filtered)**	**a.f. (Filtered)**
Paint Horse	1872	8	0	887	0.0022
***SW6***					
		**Genotypes-Unfiltered**			
**Breed**	**n (Unfiltered)**	***SW6/N***	***SW6/SW6***	**n (Filtered)**	**a.f. (Filtered)**
Paint Horse	1872	3	0	887	0.00056
Quarter Horse	4906	1	0	2793	0
Total		4	0		
**Combinations**					
		**Genotypes-Unfiltered**			
**Breed**	**n (Unfiltered)**	***SW1/SW6***	***SW1/N* + *SW2/N***	***SW1/SW1* + *SW2/N***	***SW1/N* + *SW2/SW2***
Paint Horse	1872	1	14	1	0
Quarter Horse	4906	0	61	8	3
Total		1	75	9	3

Only the combinations of splashed white variants detected in the dataset are presented herein.

## Data Availability

These data will be made available upon reasonable request to the corresponding author.

## Supplementary Materials

The following supporting information can be downloaded at https://www.mdpi.com/article/10.3390/genes13091641/s1, Table S1: Number of individuals tested per breed before and after filtering for relatedness. Table S2: Genes, reported alleles, and variant descriptions for the alleles investigated in this study. Table S3: Genotype counts for eact coat color locus analyzed in this study across breeds. Table S4: Allele frequencies for each coat color locus analyzed in this study after filtering for relatedness across breeds. References [41,42] are cited in the supplementary materials.
